# Image-Processing-Based Subway Tunnel Crack Detection System

**DOI:** 10.3390/s23136070

**Published:** 2023-06-30

**Authors:** Xiaofeng Liu, Zenglin Hong, Wei Shi, Xiaodan Guo

**Affiliations:** 1 School of Land Engineering, Chang’an University, Xi’an 710054, China; lxfgxd@163.com; 2 Shaanxi Province Institute of Geological Survey, Xi’an 710054, China; gxdgeo@163.com; 3 Shaanxi Hydrogeology Engineering Geology and Environment Geology Survey Center, Xi’an 710068, China; shiwei_gz@163.com; 4 Shaanxi Engineering Technology Research Center for Urban Geology and Underground Space, Xi’an 710068, China

**Keywords:** subway tunnel, crack detection, image processing, Alexnet algorithm

## Abstract

With the increase in urban rail transit construction, instances of tunnel disease are on the rise, and cracks have become the focus of tunnel maintenance and management. Therefore, it is essential to carry out crack detection in a timely and efficient manner to not only prolong the service life of the tunnel but also reduce the incidence of accidents. In this paper, the design and structure of a tunnel crack detection system are analyzed. On this basis, this paper proposes a new method for crack identification and feature detection using image processing technology. This method fully considers the characteristics of tunnel images and the combination of these characteristics with deep learning, while a deep convolutional network (Single-Shot MultiBox Detector (SSD)) is proposed based on deep learning for object detection in complex images. The experimental results show that the test set accuracy and training set accuracy of the support vector machine (SVM) in the classification comparison test are up to 88% and 87.8%, respectively; while the test accuracy of Alexnet’s deep convolutional neural network-based classification and identification is up to 96.7%, and the training set accuracy is up to 97.5%. It can be seen that this deep convolutional network recognition algorithm based on deep learning and image processing is better and more suitable for the detection of cracks in subway tunnels.

## 1. Introduction

With the continuous development of urban subway construction and the continued existence of tunnels constructed even earlier, various types of diseases have appeared in subway tunnels [1,2]. Due to the low tensile strength of concrete, small cracks are generally generated on the surface of the concrete. Such small cracks do not affect the mechanical properties of the tunnel and are, therefore, classed as harmless cracks [3]. However, under the action of temperature change, foundation settlement, steel corrosion, and other factors, the development and interconnection of microcracks can eventually lead to a deterioration in performance, and, thus, it is necessary to strengthen our detection of these cracks [4]. It is difficult to identify the initial characteristics of early tunnel cracks. If we can find such cracks early and take reasonable and effective preventive measures, this would greatly reduce the difficulty in later maintenance work and reduce maintenance costs. Therefore, it is necessary to intensify the work regarding the operation, maintenance, and inspection of tunnels to improve their overall safety level [5].

The widespread use of image processing technology in various detection fields is the result of the continuous advancement of computer technology, which can solve some of the past shortcomings of traditional detection [6,7]. The ability to use image processing technology for long-distance, noncontact crack identification not only protects the safety of workers but also prevents damage to the tunnel itself. Therefore, it is crucial to study and develop a crack detection system combined with this image processing technology by utilizing the characteristics of tunnel images. This article proposes a subway tunnel crack recognition system based on methods such as grayscale erosion, block contrast, stretching, and binarization to improve image contrast and quality. Due to the high grayscale values of the background area and the low grayscale values of the crack area, the difference in grayscale values between two regions is used to classify image pixels. By maximizing the variance between classes, the system automatically calculates the threshold and updates it based on the segmentation effect. The modified threshold can effectively identify cracks in the image background, maximize the protection of crack information, and correct the noise sensitivity defects of edge detection operators. It also maintains the enhanced characteristics of cracks to better detect them.

## 2. Related Work

Cracks in subway tunnels are ubiquitous and one of the causes of tunnel diseases. Cracks in subway tunnels not only affect their appearance but also lead to dangers in terms of their carrying capacity and durability, and can even bring about large economic losses and societal safety hazards in severe cases. Yuan W indicated that the detection and investigation of tunnel cracks in subway tunnels are special inspection items [8]. Xue F’s research found that the components in different positions of the tunnel are repeatedly loaded and unloaded during the operation [9]. Huang H pointed out that various researchers have developed tunnel detection equipment [10]. Bao X studied the seismic performance of large subway tunnel structures in liquefied soil layers and analyzed the effect of mitigation methods for different steel bar thicknesses on reducing cracks [11]. Lee H indicated that during the construction of subway tunnels, the construction process of side rails should be considered, and a stability assessment should be conducted [12]. However, the early detection of tunnel cracks is difficult to achieve during the actual detection process using theoretical knowledge alone. In addition, due to the harsh environment of the tunnel and insufficient lighting, crack detection becomes more difficult.

Due to the gradual maturing of image processing technology, scholars have increasingly been studying the technology and applying it in different industries. At present, the detection of cracks in subway tunnels using this technology has also attracted much attention. Wang R showed that in the image processing of crack detection systems, false information and breakpoint problems required more efficient threshold segmentation and breakpoint connection algorithms [13]. Dai Z said that the detection of tunnel cracks has become the main method in this field to overcome the shortcomings of missed detection [14]. Based on crack detection using a single image, Chen F C found a new fusion method to integrate the extracted information to enhance the overall performance and robustness of the system [15]. Guo C showed that fractures with different degrees of fracture create great difficulties for the extraction of subsequent fracture identification and image processing information [16]. Image processing controls the detection accuracy within a certain range and can quickly obtain crack data. It prevents interference by technicians due to the use of factors beyond their personal control and facilitates an objective assessment of the structural safety of the tunnel. Based on the above analysis, it is clear that developing an advanced tunnel crack detection system is urgently needed.

## 3. Related Algorithms Based on Image Processing

### 3.1. Subway Crack Detection Method and System Design

(1) Analysis of traditional manual detection and automated detection methods

**Figure 1:**
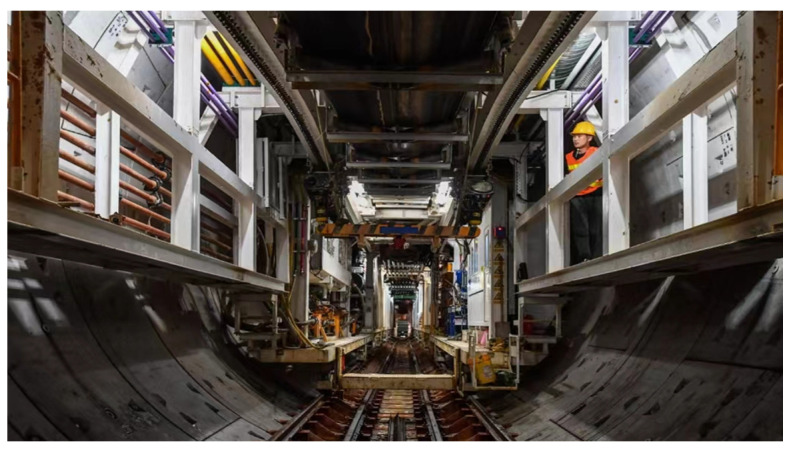
Manual inspection of a subway tunnel.

The technology for the early detection of subway tunnel cracks is not yet fully mature, and manual detection is the main method used. In recent years, many experts and scholars have been exploring and researching new technologies, and they have gradually created many new methods. According to the degree of complexity and mechanization, tunnel crack detection is divided into traditional manual detection and automatic detection [17]. Traditional manual inspection mainly relies on the detection of cracks by staff; however, there are subjective factors, and it is difficult to ensure the accuracy of the inspection results. Therefore, the traditional manual detection method requires a large amount of manpower, and, even then, the efficiency is not high. The application of the traditional manual detection method in a tunnel is shown in Figure 1.

**Figure 2:**
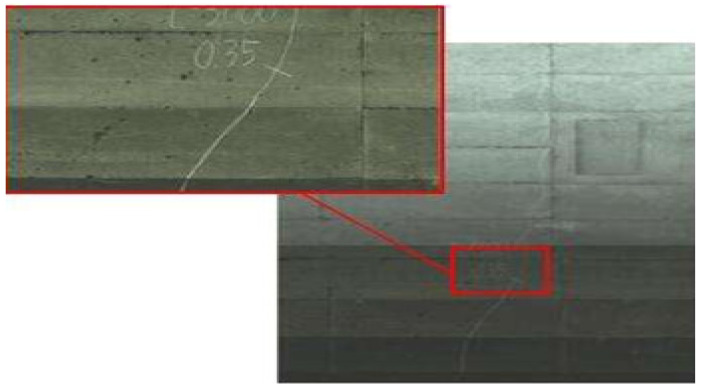
A crack map created by scanning with an automated detection method.

As can be seen from Figure 1, manual detection is suitable for detecting structures with relatively small volumes and sizes, but not for large-scale structure detection [18,19]. In the traditional tunnel crack detection process, the tunnel must be manually inspected after shutdown. For subway systems in busy cities, such detection can only be carried out at night. However, there are many problems and hidden dangers in this intermittent manual detection at night: there is insufficient light in the subway tunnel, which affects the accuracy of visual detection. In addition, manual exploration relies on the subjective judgment of the staff and is, therefore, prone to exploration omissions and judgment errors. Similarly, the speed of manual exploration is slow, the exploration process is still dangerous for personnel, and the labor cost is high. With the rapid development of computer technology, an automatic crack detection system based on image processing technology has a certain application prospect. The automatic detection method uses image processing technology to identify and classify crack information. This method overcomes the shortcomings of traditional manual detection such as low efficiency and poor accuracy, and can automatically provide detection results. An example of a crack map created by scanning with the automated detection method is shown in Figure 2.

The automatic detection method comprehensively collects various images of the tunnel and then analyzes the collected images to obtain the relevant parameters of the crack. In the example shown in Figure 2, the section was scanned using laser technology, resulting in a complete image [20].

(2) Overall design scheme of the detection system

**Figure 3:**
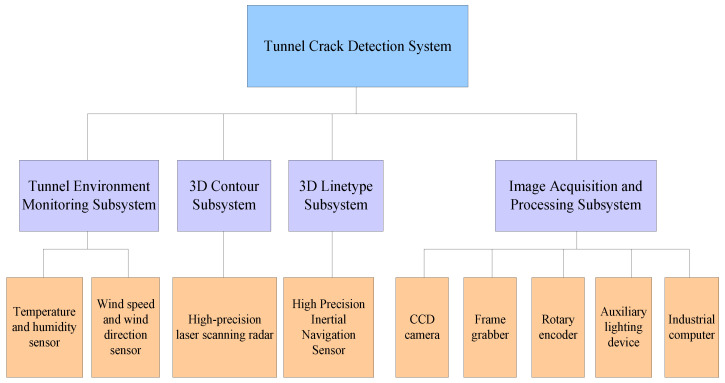
The composition of the subway tunnel crack detection system.

The subway tunnel crack detection system needs to not only detect cracks but also perform real-time detection of temperature, humidity, wind speed, wind direction, etc., as well as detect the three-dimensional direction of the tunnel through lidar and inertial navigation sensors [21]. The composition of the subway tunnel crack detection system is shown in Figure 3.

**Figure 4:**
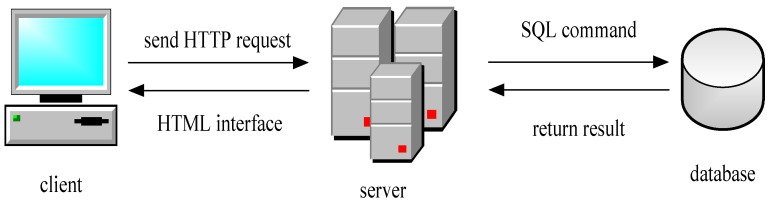
Schematic diagram of B/S mode.

After processing the collected cracks, the detected cracks are marked and the features are extracted, and finally, they are input into the database, which is also convenient for data management [22]. Data management is in the form of a management information system based on the browser/server (B/S) mode. The staff can access the server through the subway intranet to log in to the management platform, where they have corresponding permissions and can process data according to these permissions [23,24]. The client is unified with the B/S mode and the core part of the system function realization is concentrated on the server, which simplifies the development, maintenance, and use of the system. The schematic diagram of the B/S mode is shown in Figure 4.

As shown in Figure 4, the model can display, add, delete, modify, and check data, among other things, and compare them with previous data to predict trends [25].

### 3.2. Tunnel Crack Image Detection Algorithm

(1) Image preprocessing algorithm

The acquired image is a three-channel image, and the three primary color (RGB) graphics are composed of three color channels. The RGB color mode is the color standard in the industry. It obtains various colors by changing the red (R), green (G), and blue (B) color channels and superimposing them onto each other. RGB represents the red, green, and blue channels. This standard includes almost all colors that human vision can perceive, and it is one of the most widely used color systems. The three-channel information is used when the pixels are processed and turned into grayscale images [26,27]. The process of converting a three-channel image into a grayscale image is shown in Formula (1):

(1) Y(m,n)=R(m,n)×0.31+G(m,n)×0.59+B(m,n)×0.12

where Y(m,n) is the pixel value of the converted grayscale image point (m,n), and R(m,n), G(m,n), and B(m,n) are the values of the three color channels of, *R*, *G*, and *B*, respectively, at the midpoint of the three-channel image.

An open operation and a close operation are constructed via an erosion expansion combination to eliminate isolated noise points and overlap small images, respectively. Macroscopically, the erosion operation can melt the edge of the object and make the crack target texture in the image thinner. Therefore, the algorithm first performs grayscale transformation and the erosion operation on the fracture image to filter the scattered noise and the burr on the fracture edge. Let the structuring element of a grayscale image Y(m,n) be tf. The process of tf eroding Y(m,n) is recorded as Y(m,n)Θtf, and the specific definition is shown in Formula (2):

(2) $$q(m,n)=Y(m,n)\Theta tf=\left\{W/{\left(tf\right)}_{w}\subseteq Y(m,n)\right\}$$

where q(m,n) is the image showing grayscale erosion. The entire image Y(m,n) must be traversed by the structural component tf. The image q(m,n), after the erosion process, is a collection of pixel points composed of W points with the same properties. If the origin is moved to the W point, the origin of the structural component tf can be completely contained in the image Y(m,n).

**Figure 5:**
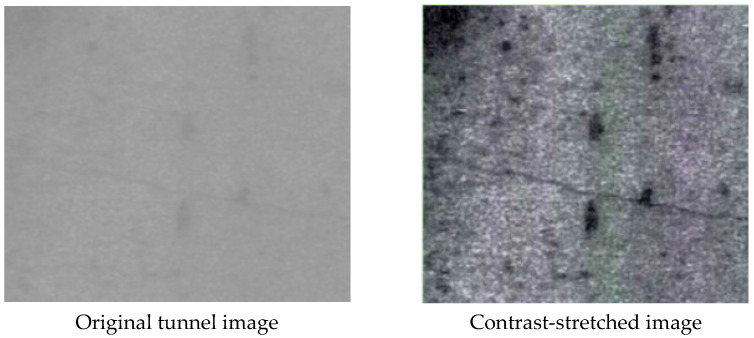
Contrast images before and after contrast stretching.

There are two types of grayscale transformations: linear stretching and nonlinear stretching. Linear stretching changes the pixel value of the image by adjusting the slope and intercept of the linear transformation function, thereby transforming the grayscale value range of the original image to another limited range. The linear stretching process is shown in Formula (3):

(3) $$h^{\prime }(a,b)=x\times h(a,b)+y$$

where h(a,b) stands for the original image, $h^{\prime }(a,b)$ is the linear stretched image, and x and y are the slope and intercept, respectively. The contrasting images before and after contrast stretching are shown in Figure 5.

As shown in Figure 5, the brightness of the image area is uneven, and this results in the cracks in the region having high and low contrast, which is not conducive to subsequent crack feature extraction. In order to expand the grayscale distribution of the image, a nonlinear transformation is required. Nonlinear transformation refers to the use of nonlinear functions such as logarithmic transformation, power transformation, exponential transformation, and piecewise function transformation to stretch the captured image. This causes the image grayscale value distribution to be more in line with human observation habits. The process is shown in Formula (4).

(4) $$p(a,b)=1/1+\left\{n/{\left[h(a,b)+eps\right]}^{\wedge }V\right\}$$

where p(a,b) is the pixel value at point (a,b) after contrast stretching, and n is the average value of the original image pixels.

(2) Image multilevel feature analysis algorithm

Rectangularity is a characteristic measure that can be used as a cohesion test [28] because it refers to the number of adjacent rectangles that an image fills in a relevant area. In order to further filter the noise components, the algorithm designs a hierarchical feature analysis model of the connected domain of the subway tunnel image based on the ideas of being multilevel and multiscale. The original tunnel image is divided into different connected regions by using the image segmentation idea, and the connected regions are analyzed with targeted stratum-level features. This filters out noise interference from, for example, non-cracks as much as possible, retains and strengthens crack features, and lays a foundation for subsequent crack image feature training and recognition detection. Speckle noise in subway tunnel images has a wide area and a high distribution density. Meanwhile, cracks are long and narrow, which makes it possible to distinguish them using the rectangularity of the connected region. The rectangularity of the connected region is calculated with Formula (5):

(5) $$B_{q}=\frac{\sum {}_{a}\sum {}_{b}\left[1-Y(a,b)\right]}{T_{q}}=\frac{M_{q}}{T_{q}}$$

where $M_{q}$ is the zero-order rectangle of the connected area, and the filter assumes that the image $L_{x}(a,b)$ has been filtered by the zero-order moment texture feature of the first-layer connected area $Y_{q}(a,b)$. As with the previous layer of filtering, the noise component is then extracted from the image. Assuming that the number of retrieved connected regions is $M_{B}$ and the rectangularity threshold is $S_{b}$, the calculation to obtain the speckle noise $l_{y}(a,b)$ is as follows:

(6) $$l_{y}(a,b)=\left\{Y_{q}(a,b)\left|B_{q}>S_{b},q=1,2,\ldots ,M_{B}\right.\right\}$$

Most of the noise elements in the image have been removed after filtering. Filtering based on the rectangularity of the connected area can filter out most of the speckle noise in the subway tunnel image and retain the elongated crack area. At this time, most of the noise areas in the image have been filtered out, but some large block noise areas connected to the fracture skeleton or with low cohesion have not been filtered out, and thus further operations are needed. The algorithm carries out shape extremum filtering based on the connected region by combining the maximum width $v_{\max}$, the maximum length $u_{\max}$, and the rectangle $B_{q}$ of the connected region. After two stages of filtering, the crack image $l_{y}(a,b)$ is kept alone, and the relevant regions are extracted again to avoid repeating the calculation process. Let the total number be $M_{v}$ and the extracted correlation region be $D_{q}(a,b)$, then, Formula (7) represents the extended extraction process of the large-area block noise $l_{d}(a,b)$.

(7) $$l_{d}(a,b)=\left\{D_{q}(a,b)\left|v_{\max}>S_{v},u_{\max}>S_{u},B_{q}>S_{B},q=1,2,\ldots ,M_{v}\right.\right\}$$

Connected regions that all exceed the threshold are called extended large-area block noise regions. These include a connection area width threshold $S_{v}$, a height threshold $S_{u}$, and a rectangle threshold $S_{B}$. However, it should be noted that there are still large-area areas of lumpy noise with low cohesion caused partly by uneven tunnel lining and thickness. Both the zero-order moment and the squareness of this type of noise are large, making it indistinguishable from a fracture region with a short length.

(3) Circumscribed rectangle image extraction of feature texture connected area

In order to retain all crack regions, non-crack-based types of noise such as wall scratches and water stains in tunnel images are filtered out as much as possible through image preprocessing and layered feature analysis. To this end, before the extraction of the original image corresponding to the connected area, the length–width ratio can be set artificially according to the original image proportions, experimental experience, and crack texture characteristics, and the minimum circumscribed rectangle can be appropriately expanded to ensure that the size proportions of the extracted image are the same or close to the original image and reduce the subsequent image recognition error. The smallest bounding rectangle of the connected area package $C_{q}(a,b)$ of the reserved part of the image and its coordinates $\left(a_{\min},b_{\min}\right)$, $\left(a_{\min},b_{\max}\right)$, $\left(a_{\max},b_{\min}\right)$, and $\left(a_{\max},b_{\max}\right)$ can be determined. The formula for calculating the aspect ratio D:P of the minimum circumscribed rectangle of the connected region is shown in Formula (8).

(8) $$D:P=\frac{column}{row}=\frac{a_{\max}-a_{\min}}{b_{\max}-b_{\min}}$$

The principle of expanding the size of the rectangle circumscribed by the longitudinal crack is shown in Formulas (9) and (10):

(9) $$b_{\min}=\max\left\{1,b_{\min}-0.6\times \left[0.74\times (a_{\max}-a_{\min})-(b_{\max}-b_{\min})\right]\right\}$$

(10) $$b_{\max}=\min\left\{1,b_{\max}+0.6\times \left[0.74\times (a_{\max}-a_{\min})-(b_{\max}-b_{\min})\right],375\right\}$$

Therefore, when the circumscribed rectangle corresponds to the original image extraction, the minimum circumscribed rectangle is appropriately expanded. While filling the crack area with images, this process ensures that the aspect ratio of the image is as close as possible to the original image and avoids the error caused by the large size difference in the subsequent image classification.

### 3.3. Algorithm Design of Tunnel Crack Identification and Feature Detection

(1) Tunnel complex image target detection algorithm

Considering the advantages and disadvantages of image recognition and feature detection algorithms for subway tunnels, an SSD algorithm is developed to detect objects in complex images. The main network structure used by the SSD method is VGG16. The estimated bounding box size ratio $t_{q}$ for each feature map is calculated with Formula (11):

(11) $$t_{q}=t_{\min}+\frac{t_{\max}-t_{\min}}{n-1}(q-1),q\in \left[1,n\right]$$

**Figure 6:**
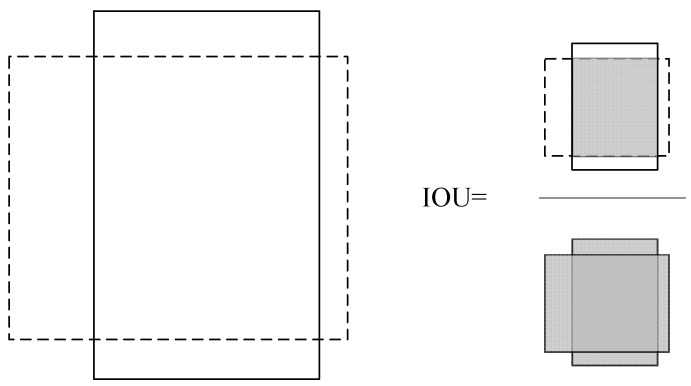
Object detection IOU calculation.

The expected bounding box output of each feature map is convolved with two convolution kernels, and the program compares it to the actual label value box. When the intersection-over-union ratio (IOU) exceeds a predetermined threshold, matching target detection occurs. Unlike classification and recognition, target detection needs to judge the position and category of the target in the image at the same time. Therefore, there are great differences in the evaluation methods. The evaluation target is not the whole image, but the prediction boundary box generated in the process of network training and that participates in the loss function calculation and forward and backward propagation. The target detection IOU calculation is shown in Figure 6.

For each type of target, if there is a target sample for this category in the prediction boundary box and the *IOU* is greater than the set threshold, it is a positive sample. Conversely, if the target category of the sample in the prediction boundary box is wrong or the *IOU* of the target sample for this category is less than the set threshold, it is a negative sample. Figure 6 illustrates how the multiobject detection stage uses the set *IOU* threshold to evaluate the location of the algorithm’s expected bounding box, calculated as:

(12) $$IOU=\frac{P_{1}\cap P_{2}}{P_{1}\cup P_{2}}$$

where $P_{1}$ is the area of the bounding box predicted by the algorithm, and $P_{2}$ is the area of the ground-truth label value box. According to the actual situation with the subway tunnel image, the target category to be recognized is selected, and the real label database is created after marking the target category and position in the image.

(2) Image classification and recognition algorithm for tunnel cracks

Local response normalization (LRN) completes the data normalization operation in standard deep convolutional networks, as it is used to improve the generalization ability and training speed of convolutional neural networks. Batch normalization (BN) is used instead of LRN in the upgraded Alexnet deep convolutional network to perform standard data normalization operations. In this Alexnet deep convolution network, a self-learning process is completed through forward propagation and backward propagation. Through forward propagation from the bottom to the top, the output value from the calculation results of the network model is obtained through a layer-by-layer calculation. This speeds up the learning rate of the network and prevents the intermediate layers from changing during training on the image data. This makes it suitable for extracting image features from subway tunnels. For the feature extraction, classification, and identification of cracks in subway tunnel photos, this method uses a lightweight open-source deep learning framework (Caffe) to build an upgraded Alexnet deep convolutional network.

(13) $$m_{i}=t(v_{i})$$

(14) $$v_{i}=\omega_{i}m_{i-1}+d_{i}$$

where the activation functions t and $m_{i}$ are the output of the i layer, $m_{i-1}$ is the output of the i−1 layer, $\omega_{i}$ is the weight value of the i layer, and $d_{i}$ is the bias. Therefore, the calculated gradient of backpropagation is:

(15) $$\varsigma_{y}=\frac{\partial W_{y}}{\partial v_{y}}$$

(16) $$W_{y}=f_{y}-n_{y}$$

(17) $$\frac{\partial W_{y}}{\partial d_{i}}=\frac{\partial W_{y}}{\partial v_{y}}\frac{\partial v_{y}}{\partial d_{i}}=\varsigma_{y}$$

(18) $$\frac{\partial W_{y}}{\partial \omega_{i}}=\frac{\partial W_{y}}{\partial v_{y}}\frac{\partial v_{y}}{\partial \omega_{i}}=\varsigma_{y}m_{i-1}$$

where $\varsigma_{y}$ represents the rate of change of the error $W_{y}$ relative to the output $v_{y}$, and the sample error $W_{y}$ is the difference between the label value $f_{y}$ of the sample and the network output value $n_{y}$. The rate of change of the sample error relative to the deviation $d_{i}$ and weight value $\omega_{i}$ can be calculated. The following formula represents the weight value update rules:

(19) $$\omega_{l+1}=\omega_{l}+u_{l+1}$$

where l is the number of iterations and u is the momentum value.

The SVM achieves the goal of data classification by maximizing the distance between positive and negative data values based on the concept of structured risk minimization. The SVM uses the hinge loss function to calculate the empirical risk and adds the regularization term to the solution system to optimize the structural risk. It is a sparse and robust classifier. The SVM can be used for nonlinear classification via the kernel method, which is one of the common kernel learning methods. A three-level cascaded SVM classifier can be constructed, where the first two cascaded SVM classifiers are used to gradually predict and score, and then the third-level SVM classifier is used for target confirmation to improve data accuracy [29,30]. When combined with real images of subway tunnels, feature data for the aspect ratio of the largest connected area can be derived. The ratio between the maximum values $p_{\max}$ and $e_{\max}$ of the length and width of the connecting region is called the aspect ratio k of the largest connecting region, as shown in Formula (20).

(20) $$k=\frac{p_{\max}}{e_{\max}}$$

### 3.4. Selection of Model Building Environment and Hardware Equipment

(1) Selection of model building environment

The crack detection system is designed for subway tunnel detection, with the aim of identifying cracks arising due to different types of tunnel defects. In order to ensure the efficient performance of the crack detection system in terms of safety, the entire system needs to be installed on a rail vehicle that can provide structural stability for the equipment. Due to the negative impact of the low light intensity in the tunnel on obtaining high-quality images, it is necessary and important to equip the camera with sufficient lighting equipment. The system can only enter the subway tunnel within the maintenance window, and the operating time is only about 2 h. Therefore, the system needs to obtain a large number of tunnel images in a limited time and accommodate a large amount of image data obtained through a single inspection. Considering the actual situation of the tunnel and the cost-effectiveness of hardware equipment, the selection of the system’s main components is discussed in this section.

(2) Selection of hardware equipment

For the choice of hardware, CMOS cameras have attracted great interest in high-speed image processing due to their low power consumption and fast image reading speed. Line-scanning cameras capture images through linear scanning, but due to insufficient lighting, the captured images are prone to being of low quality. Common lighting devices such as flashlights are not suitable for line-scanning cameras. Laser light sources have a high illumination intensity, which can ensure the uniformity of image brightness. Therefore, the high-speed industrial line-scanning CMOS camera Basler racer ral6144 was chosen for image acquisition, and a customized laser light source was selected for illumination. The captured images were transmitted to the computer via an image capture card.

To ensure that the captured image contains information about half of the tunnel surface, three cameras and two laser light sources were evenly spaced on the frame, with a spacing of 30° between each device. The image processing system can control the image acquisition system to obtain images of the tunnel surface, store a large number of captured images, and perform image data analysis. An industrial computer equipped with image acquisition control software and image analysis software was used for real-time image acquisition and offline image processing. A small and powerful portable i7 processing industrial computer was used for this.

## 4. Crack Detection Experiment Based on Image Processing

### 4.1. Image Grayscale Transformation Experiment

**Figure 7:**
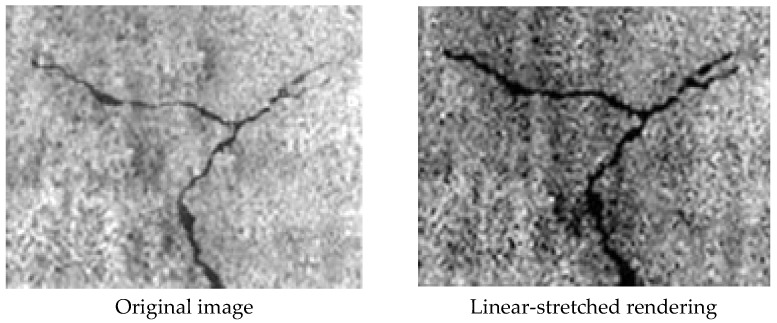
Comparison of an original image and a linear-stretched rendering.

Because nonlinear stretching can only expand or compress the regions with low or high grayscale values, it is not generally suitable for the grayscale transformation of crack images. Therefore, this paper mainly compares linear stretching and histogram equalization. The comparison between the original image and the linear stretching effect is shown in Figure 7.

**Figure 8:**
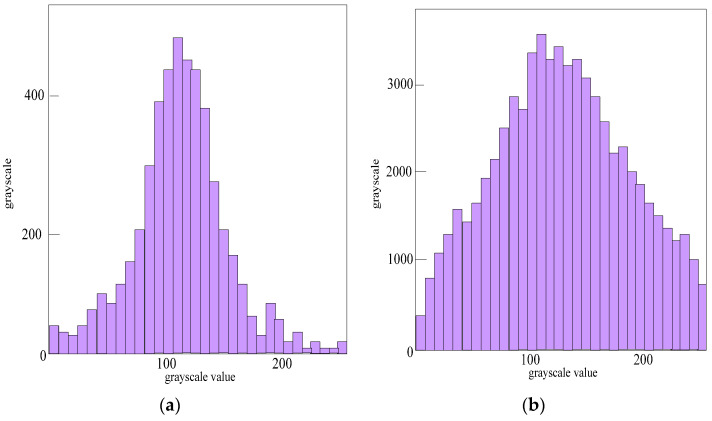
Comparison of original histogram and linear-stretched histogram. (**a**) Original histogram. (**b**) Linear-stretched histogram.

It can be seen from Figure 7 that the grayscale values of the original image are not clear and it is difficult to capture fine cracks, but after histogram equalization, the contrast between the crack image and the background image is significantly enhanced. The corresponding histogram is shown in Figure 8.

**Figure 9:**
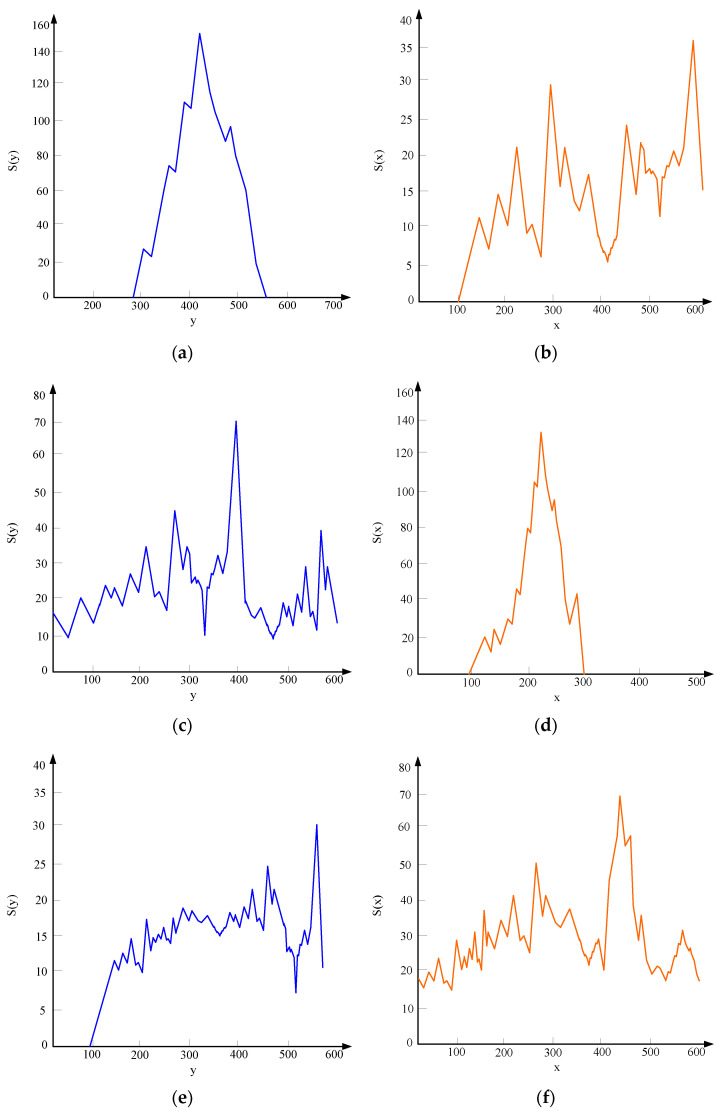
Projections of transverse cracks, longitudinal cracks, and oblique cracks in the horizontal and vertical directions. (**a**) The horizontal projection of the transverse cracks. (**b**) The vertical projection of the transverse cracks. (**c**) The horizontal projection of the longitudinal cracks. (**d**) The vertical projection of the longitudinal cracks. (**e**) Horizontal projection of the oblique fissures. (**f**) Vertical projection of the oblique fissures.

It can be seen from Figure 8a that the grayscale values of the original crack image are mainly concentrated in the grayscale value range of 90–150, the fluctuation is large, and other grayscale areas are not widely covered. As can be seen from Figure 8b, due to the linear stretching, the grayscale values of the image evenly cover the entire grayscale range of 20–260. After the comparison based on grayscale experiments, the projection characteristics of the cracks are then compared. In this paper, the fracture projection was drawn in the coordinate system, and the projection curve was obtained. Projection results for transverse cracks, longitudinal cracks, and oblique cracks in the horizontal and vertical directions are shown in Figure 9.

It can be seen from Figure 9a that the transverse cracks have an obvious peak value in the horizontal direction, and the fluctuation range of the value is large. As Figure 9b shows, the projected distribution of transverse cracks in the vertical direction is uniform and gentle. From Figure 9c, it can be seen that the longitudinal cracks are directional and the projection distribution is relatively uniform. Figure 9d shows that the longitudinal cracks are opposite to the transverse cracks and have obvious peaks. While Figure 9e shows that the oblique cracks do not have large fluctuations in the horizontal direction. Finally, it can be seen from Figure 9f that the projection of the oblique cracks in the vertical direction is relatively gentle, and the projection in the horizontal direction is broadly similar.

### 4.2. Image Target Detection Experiment

Using deep learning, this paper constructs an SSD deep convolutional neural network and verifies and analyzes it, as shown in Table 1.

As shown in Table 1, on the basis of the sample library test, 9171 actual images of subway tunnels and 21,547 target real-label value boxes are obtained by adopting the balanced improvement method. The training set contains 7130 images, the validation set contains 1020 images, and the test set contains 1021 images. After the training, the model generated by the training set is used to detect the tunnel image directly obtained by the detection system. Different confidence thresholds can be set, and prediction boundaries that meet different score requirements can be obtained. In the actual regular inspection of subway tunnels, the focus is on whether cracks can be found to determine the areas of concern. That is, it tries to ensure that all objects are detected. The detection rates of cracks under different confidence threshold ranges are shown in Table 2.

Under the confidence level of 0.1, a total of 1240 cracks were detected, with a detection rate of 92.96%. At the confidence level of 0.5, 878 cracks were detected, and the detection rate was 64.38%. Therefore, in the detection results, especially for small cracks, the repeated box selection of multiple prediction boxes is prone to occur, but this does not affect the overall detection effect. This method needs to be perfected in future research, and the recognition accuracy for complex images in subway tunnels can be improved by further fusion.

### 4.3. Experimental Results of Image Classification and Recognition

**Figure 10:**
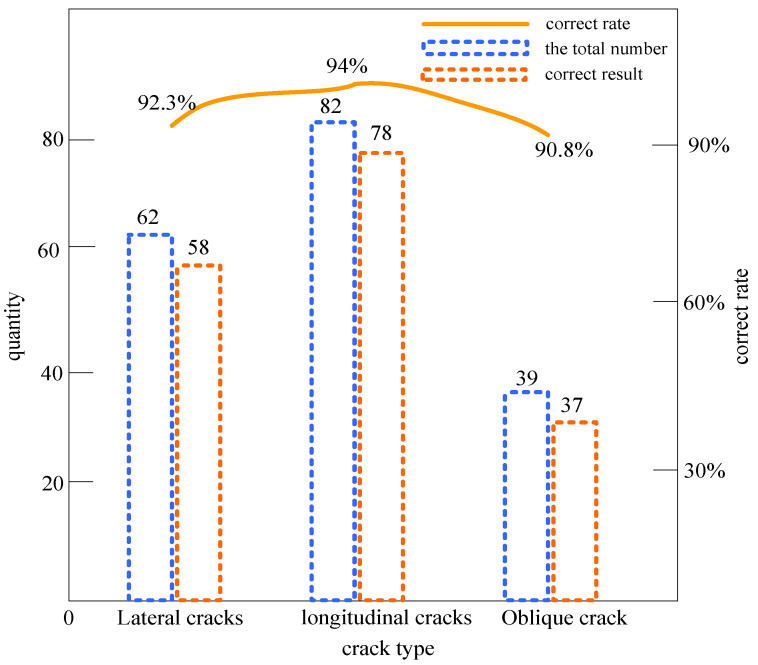
Experimental results of fracture classification.

Due to the difficulty in identifying crack images in subway tunnels, the function was continuously adjusted according to the experimental results during the experiment to improve efficiency and accuracy. In this experiment, a total of 3000 data images were selected, the data images were extracted with features, the output data were normalized, and then the output data were read and trained. Because the initial weights were random, the error in the result was also random, and the training result taken was the best training result selected from multiple experiments. The experimental result can be obtained as long as some output(s) approaches 1 and other outputs approach 0, as then the fracture type can be determined. The experimental classification results are shown in Figure 10.

The experimental results show that the detection accuracy with transverse cracks reached 92.3%; the detection accuracy with longitudinal cracks reached 94.0%; and the detection accuracy with oblique cracks reached 90.8%. The output results of the analysis can be obtained for transverse cracks and longitudinal cracks with regular shapes, because, even if they have a certain angle in the horizontal or vertical direction, this would not affect the results of the system judgment, and at the same time, a high classification accuracy can be achieved. This verifies the rationality of feature selection. However, considering that not all cracks are regularly shaped, some misclassified crack images were found during the experiment. Because of the distortion and ductility of the cracks in these images, it is difficult to define their type manually, and the analysis of the misclassified images shows that it is this type of crack image that interferes with the judgment results. At the same time, it can be seen that the set of eigenvectors selected in the experiment can classify and identify cracks well, and the average accuracy rate of the system for the identification of the three types of cracks reached 92.4%.

Using the original image sample library for classification and recognition, the training accuracy was 74.7% and 69.2%, respectively. By combining the feature texture after pixel processing with the improved bounding rectangle correspondence, a better sample library was obtained. The training accuracy reached 87.8%, and the test accuracy reached 88%. At the same time, a comparison experiment between subway tunnel image classification and recognition was carried out with the improved Alexnet deep convolutional network, and the experimental results are shown in Table 3.

As can be seen from Table 3, the model was classified and identified based on the Alexnet deep convolutional neural network. The results show that the training accuracy and test accuracy of the method were 87% and 88.2%, respectively. Compared with the original image, the training accuracy of the binarized image sample library was 79.6%. This approach uses the method corresponding to the minimum circumscribed rectangle of the feature texture connection area after pixel processing to establish a new sample library. The sample library has the characteristics of completeness, large proportions, and extractability. The method has a training accuracy of 94.3% and a test accuracy of 93%. The main reason for this is that, after batch preprocessing and hierarchical feature analysis, the tunnel image was converted from a three-channel image to a grayscale binary image, the content information was reduced, and the features that can be extracted during classification and recognition were reduced. It is difficult to distinguish cracks from pseudo-cracks or small cracks from noise by using the pixel information contained in the grayscale binary image. On this basis, the extracted original image was matched with the improved circumscribed rectangle to make its size broadly the same. This reduces the impact of network feature extraction and classification prediction, achieving a 97.5% training accuracy and 96.7% testing accuracy. This classification and identification algorithm is fast and accurate and cannot be affected by human subjective factors. It can be directly used for real image detection in subway tunnels.

## 5. Conclusions

(1) With the aim of identifying complex crack images in subway tunnels, a deep-learning-based SSD deep convolutional neural network method was proposed.
(2) In the experiment, original images and linear-stretched effect maps, as well as the horizontal and vertical projection results, of transverse cracks, longitudinal cracks, and oblique cracks, were compared. In order to verify the application of this method in subway tunnels, an SVM-based classification and identification method was adopted.
(3) Experiments show that the method combining image processing and deep learning has better performance than the SVM-based classification and recognition method.
(4) There are still several issues that need to be resolved because of the complicated internal environment of subway tunnels, the numerous interference elements, and the constrained number of image samples. Therefore, the algorithm still needs to be optimized and improved, and the scientific basis and rigor of the experiment should be strengthened in future work.

## Figures and Tables

**Table 1:** The number of targets included in the sample library.

	Number of Images	Number of Cracks	Total
Training Set	7130	9730	16,860
Validation Set	1020	1350	2370
Test Set	1021	1296	2317
Total	9171	12,376	21,547

**Table 2:** Fracture detection rates under different confidence threshold ranges.

	0.1	0.2	0.3	0.4	0.5
Confidence Threshold	1240	1126	997	956	878
Crack Detection Rate	92.96%	83.65%	68.13%	66.25%	64.38%

**Table 3:** Classification and recognition accuracy of sample library based on Alexnet deep convolutional network.

	Raw Image Sample Library	Binary Image Sample Library	Feature Texture Bounding Rectangle	Improved Bounding Rectangle
Alexnet Training Accuracy	87%	79.6%	94.3%	97.5%
Alexnet Test Accuracy	88.2%	79.3%	93%	96.7%

## Data Availability

The data that support the findings of this study are available from the corresponding author.
